# Supplementary material for: Influence of Ziziphus lotus (Rhamnaceae) Plants on the Spatial Distribution of Soil Bacterial Communities in Semi-Arid Ecosystems
Source: Microorganisms. 2025 Jul 25;13(8):1740. doi: 10.3390/microorganisms13081740 (PMC12388012; doi:10.3390/microorganisms13081740)
Supplement: Supplementary file 1 [file microorganisms-13-01740-s001.zip › microorganisms-3759592-supplementary.pdf]

Supplementary Information

Table S1. Physicochemical characteristics of different *Ziziphus lotus* clusters in barley-planted and non-barley-planted fields across varying sampling distances. EC: electrical conductivity; OM: organic matter; CEC: cation exchange capacity. Cluster codes begin with 'C' for barley-planted fields, followed by the cluster number (1–5) and the sampling distance (0, 3, or 6 m). Similarly, 'T' denotes clusters from unplanted fields, followed by the cluster number (1–2) and the sampling distance.

Cluster	Clay	Silt	Sand	pH	EC	$CaCO_3$	OM	P_2O_5	K_2O	Na_2O	MgO	CaO	NO_3^-	NH_4^+	Total N	Cu	Fe	Mn	Zn	CEC
	%	%	%		ms/cm	%	%	mg/kg	mg/kg	mg/kg	mg/kg	mg/kg	mg/kg	mg/kg	%	mg/kg	mg/kg	mg/kg	mg/kg	meq/100g
C1-0	24	48	28	8.05	0.63	0.4	2.8	125	1006	189	626	5308	80.4	6.18	0.18	0.69	2.06	11.12	0.76	12
C1-3	34	44	22	8.37	0.19	0.3	2.2	124	851	231	632	5230	57.29	3.12	0.17	0.93	2.41	11.12	0.98	15.5
C1-6	32	46	22	8.69	0.13	0.3	1.66	34	466	238	567	5223	7.86	0.12	0.13	0.72	1.92	6.34	0.37	14.2
C2-0	26	48	26	8.17	0.4	0.5	2.15	67	470	253	609	6177	61.25	3.52	0.17	0.74	1.78	9.95	0.59	12.1
C2-3	28	50	22	8.7	0.13	0.3	1.71	31	327	244	566	4563	7.17	0.16	0.13	0.7	1.82	5.92	0.44	12.6
C2-6	30	50	20	8.66	0.11	0.3	1.72	27	317	196	578	4857	3.29	0.02	0.13	0.8	2.12	7.67	0.37	13.4
C3-0	24	44	32	8.14	0.27	0.6	2.81	70	654	294	629	6070	73.49	3.07	0.18	0.68	1.77	13.51	0.74	11.8
C3-3	28	52	20	8.57	0.16	0.5	2.18	37	410	224	567	5628	12.51	0.2	0.17	0.65	1.76	6.31	0.38	12.9
C3-6	30	50	20	8.48	0.15	0.6	1.77	32	406	200	558	5308	9.52	0.05	0.14	0.8	2.11	8.79	0.39	13.2
C4-0	26	48	26	8.18	0.31	1.1	3.2	98	700	155	652	6360	63.94	4.1	0.19	0.66	1.72	13.92	0.75	12.5
C4-3	26	52	22	8.38	0.22	0.8	1.78	44	448	283	667	6687	14.91	0.83	0.14	0.76	2.1	8.79	0.51	11.4
C4-6	30	50	20	8.58	0.18	0.7	1.68	40	401	265	565	5817	12.34	1.11	0.13	0.75	2.19	7.45	0.48	13
C5-0	30	42	28	8.17	0.27	0.5	2.91	90	570	230	647	4901	60.08	1.16	0.18	0.6	1.55	9.92	0.76	14.4

C5-3	26	50	24	8.62	0.12	4	1.93	35	375	207	544	4759	9.49	0.14	0.15	0.69	1.71	6.6	0.39	11.9
C5-6	26	52	22	8.66	0.12	0.5	1.68	35	359	216	539	5661	6.75	0.06	0.13	0.68	1.65	5.92	0.35	11.6
T1-0	24	42	34	8.22	23	0.5	2.53	136	791	122	507	4797	58.21	1.31	0.17	0.87	2.11	18.61	1.06	11.6
T1-3	22	50	28	8.36	0.22	0.5	1.71	43	352	200	439	3911	30.31	0.11	0.13	0.66	1.73	5.81	0.44	10
T1-6	22	50	28	8.35	0.17	0.6	1.53	37	339	197	493	4597	12.89	4.49	0.12	0.67	1.93	5.87	0.43	9.8
T2-0	22	50	28	8.06	0.29	0.6	2.53	72	619	185	575	6556	57.08	3.04	0.17	0.68	1.76	20.11	0.59	10.8
T2-3	26	52	22	8.33	0.18	0.5	1.48	39	411	190	506	6352	40.67	0.55	0.11	0.68	1.71	5.61	0.37	11.4
T2-6	30	50	20	8.6	0.15	0.5	1.46	30	354	180	500	5238	24.54	0.06	0.11	0.76	1.96	4.69	0.42	12.9

Table S2. Results of multivariate ANOVA showing the influence of soil physicochemical properties on microbial community structure. Significance levels (*p*-values) are indicated, with asterisks denoting statistical significance (*$p \leq 0.05$, **$p \leq 0.01$, *$p \leq 0.001$).

Variable	Diversity	DF	F_value	*P*_value	Significance
Clay	Shannon	1	0.06	0.81	
Silt		1	1.42	0.24	
pH		1	2.38	0.13	
EC		1	0.11	0.74	
$CaCO_3$		1	2.23	0.14	
Organic matter		1	0.51	0.48	
P_2O_5		1	1.29	0.26	
K_2O		1	0.05	0.82	
Na_2O		1	2.96	0.09	
MgO		1	1.01	0.32	
CaO		1	0.01	0.92	
NO_3^-		1	0.12	0.74	
NH_4^+		1	0.22	0.64	
Total N		1	1.29	0.26	
Cu		1	0.54	0.46	
Fe		1	0.70	0.40	
Mn		1	0.05	0.83	
Zn		1	2.78	0.10	
CEC		1	0.00	0.95	
Clay	Simpson	1	0.58	0.45	
Silt		1	4.41	0.04	*
pH		1	1.77	0.19	
EC		1	0.01	0.91	
$CaCO_3$		1	0.94	0.34	
Organic matter		1	1.23	0.27	
P_2O_5		1	1.61	0.21	
K_2O		1	0.10	0.75	
Na_2O		1	0.72	0.40	
MgO		1	1.04	0.31	
CaO		1	0.06	0.80	
NO_3^-		1	0.52	0.48	
NH_4^+		1	0.17	0.68	
Total N		1	0.03	0.87	
Cu		1	0.01	0.93	
Fe		1	0.46	0.50	
Mn		1	0.12	0.73	
Zn		1	1.34	0.25	
CEC		1	0.16	0.69	

Table S3. Results of univariate ANOVA demonstrating the influence of soil physicochemical properties on microbial community structure. Significance levels (*p*-values) are indicated, with asterisks denoting statistically significant differences (*$p \leq 0.05$, **$p \leq 0.01$, ***$p \leq 0.001$).

Variable	Diversity	DF	F_value	*p*_value	Significance
Clay		1	0.06	0.81	
Silt		1	1.3	0.26	
Sand		1	1.11	0.3	
pH		1	3.33	0.07	
EC		1	0	0.98	
$CaCO_3$		1	0.99	0.32	
Organic matter		1	4.95	0.03	*
P_2O_5		1	1.45	0.23	
K_2O		1	1.67	0.2	
Na_2O	Shannon	1	1.78	0.19	
MgO		1	1.9	0.17	
CaO		1	0.8	0.37	
NO_3^-		1	3.13	0.08	
NH_4^+		1	2.13	0.15	
Total N		1	2.26	0.14	
Cu		1	1.49	0.22	
Fe		1	0.92	0.34	
Mn		1	1.48	0.23	
Zn		1	0.6	0.44	
CEC		1	0.15	0.7	
Clay		1	0.6	0.44	
Silt		1	4.02	0.05	*
Sand		1	4.69	0.03	*

pH	Simpson	1	6.48	0.01	**
EC		1	0.67	0.41	
$CaCO_3$		1	0.24	0.62	
Organic matter		1	9.64	0	***
P_2O_5		1	3.86	0.05	*
K_2O		1	3.07	0.08	
Na_2O		1	1.08	0.3	
MgO		1	3.11	0.08	
CaO		1	0.95	0.33	
NO_3^-		1	6.72	0.01	**
NH_4^+		1	3.11	0.08	
Total N		1	6.65	0.01	**
Cu		1	0.48	0.49	
Fe		1	1.01	0.32	
Mn		1	6.5	0.01	**
Zn		1	3.48	0.07	
CEC		1	0.04	0.85	

Figure S1.

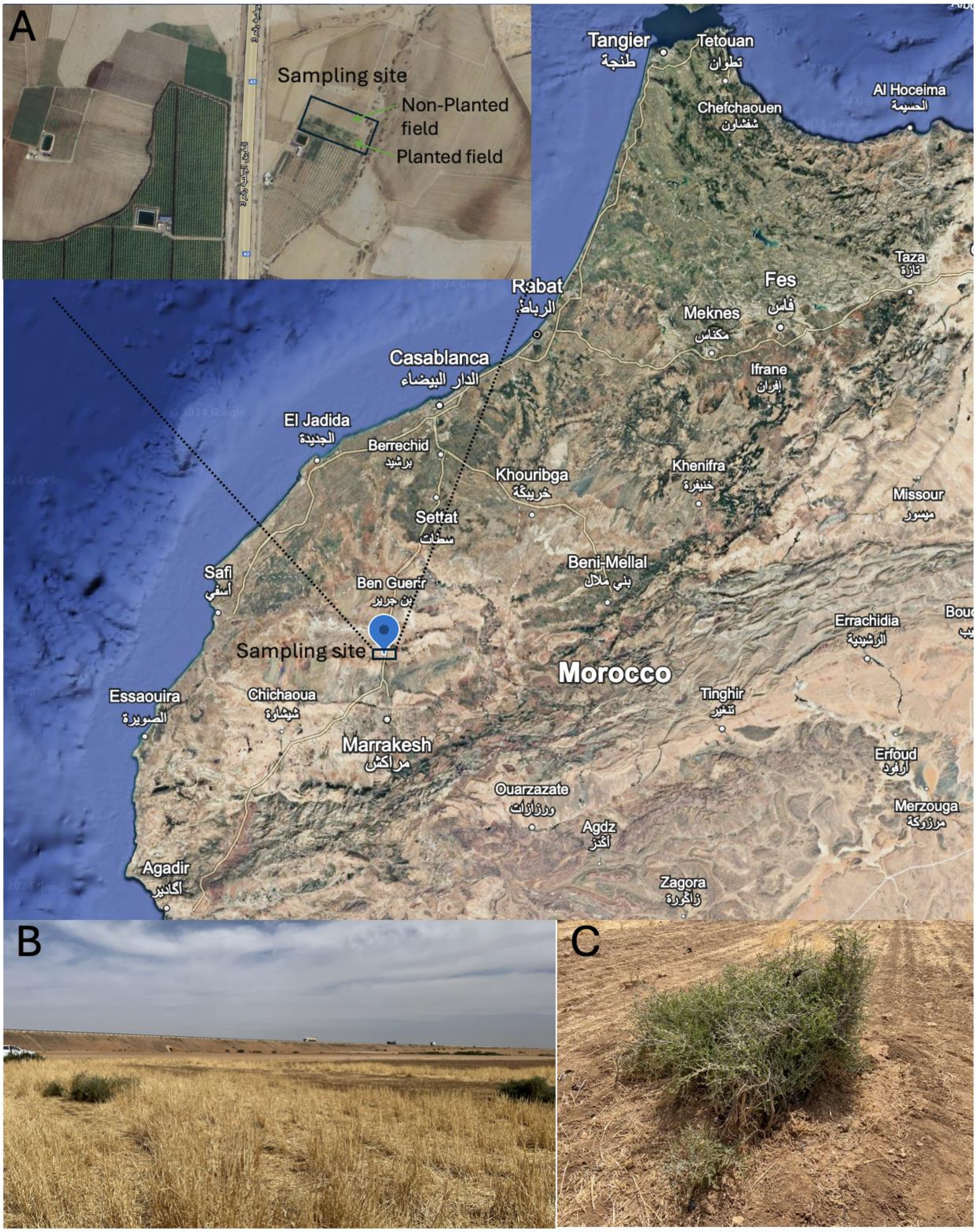

Figure S1. (A) Sampling site location in the Rhamna province, situated between Ben Guerir and Marrakech, Morocco, along the A3 highway. (B) Image depicting a barley-planted field with wild jujube shrub patches. (C) Close-up of a wild jujube shrub patch.
